# Application of Integrated BWM Fuzzy-MARCOS Approach for Coating Material Selection in Tooling Industries

**DOI:** 10.3390/ma15249002

**Published:** 2022-12-16

**Authors:** Sunil Kumar, Shubrajit Bhaumik, Lokeswar Patnaik, Saikat Ranjan Maity, Viorel Paleu

**Affiliations:** 1 Department of Mechanical Engineering, National Institute of Technology Silchar, Silchar 788010, Assam, India; 2 Tribology and Interactive Surfaces Research Laboratory (TRISUL), Department of Mechanical Engineering, Amrita School of Engineering, Amrita Vishwa Vidyapeetham, Chennai 601103, Tamil Nadu, India; 3 School of Mechanical Engineering, Sathyabama Institute of Science and Technology, Deemed to be University, Chennai 600119, Tamil Nadu, India; 4 Mechanical Engineering, Mechatronics and Robotics Department, Mechanical Engineering Faculty, “Gheorghe Asachi” Technical University of Iași, 700050 Iași, Romania

**Keywords:** coating material selection, BWM, fuzzy-MARCOS, optimization, sensitivity analysis

## Abstract

The life of metal forming dies and the efficiency of tooling industries depends on the mechanical and wear properties of tool steel. These properties can be further improved by depositing the ceramic coating on heat-treated tool steel. Numerous coating materials with various excellent features are already available commercially; however, the selection of the best coating material is still an immense challenge for users. Compared to various studies related to material selection problems in the contrasting area of utilization, remarkably, little research work has been done in tooling industries. In the present work, we have identified eight coating materials (alternatives) and nine evaluation criteria under the consultation of an expert in the tooling application and tribological field. To deal with this coating material selection problem, an integrated fuzzy-multi attributed decision-making method is proposed which comprises best worst method and fuzzy-Measurement Alternatives and Ranking according to the Compromise Solution method. This integrated fuzzy- multi attributed decision-making method is used to evaluate the alternatives, and the obtained results were scrutinized via utilizing various sensitivity analysis procedures. In the first phase of analysis, seven scenarios of criteria weight change were used, which was derived by the best-worst method; dynamic matrices are used in the second phase of analysis. In the third and fourth phases, obtained ranks were compared with those obtained by different weight calculation methods and ranking methods, respectively. In the present study, AlCrN/TiAlN coating (alternative Cm_5_) was found to be the best coating material based on the results obtained after sensitivity analysis. Further, in this study, we have proposed a novel method that helps to solve the coating material selection problem or any kind of selection complications.

## 1. Introduction

Material selection for the design and development of any die element in the tooling industries plays an important role because each die element is essential during its application. That is why tool steel has been categorized mainly into cold work and hot work tool steel. Several tool steels were developed in the past decade under the following standard: AISI, DIN, BS, JIS, AS, etc. For example, D2, O1, and A1 are cold work tool steel, and H11, H13, and H21 are hot work tool steel under AISI standards. These tool steels are heat-treated to obtain the required properties (mechanical and wear) for the particular application [1,2,3,4,5,6]; sometimes, nitriding needs to be done on the surface of heat-treated tool steels to improve the surface properties (specially wear) [7,8,9,10,11,12]. So that production can be run without any interruption in the tooling industry. The literature [6,13,14] suggested that the mechanical (hardness) and wear property of tool steel is not sufficient for the rapid and long run of production because heat-treated die elements do not have enough hardness (62 HRC or 7.88 GPa) and surface properties (wear resistance and surface roughness) to withstand unavoidable wear during rapid production. It leads to frequent maintenance (resharpening or replacement of die elements) after a few thousand of part production. Because of that, tooling industries face unintentional restrictions in the production process, machine ideal time, and maintenance time, and finally, they face a huge amount of loss. Many researchers suggested that the application of ceramic coating over heat-treated tool steels [15,16,17,18] might solve the above-mentioned problem of the tooling industry.

In this era of research on ceramic coating, a tool design engineer has countless options for choosing a coating material. The coating materials have excellent properties like hardness, elastic modulus, wear resistance, strain hardening exponent, and coefficient of thermal expansion over other alternative tool materials. This creates a coating material selection problem for the tool design engineer. Thus, the coating material selection process needs sound knowledge about the coating material related to a particular application or an experiment, such as an application in sheet metal cutting and forming, forging, plastic mold, etc. Further, experimentation is a big challenge in itself because it is a time and cost-consuming process. Not only this, but the final selection of the coating material also needs to be passed based on the result interpretation, which leads to a conflict between the tool design engineers. In this way, this study focuses on the crucial issues during the coating material selection:

- What factors and their weightage should be considered when selecting the best coating material and the evaluations of experts who are highly experienced in the coating application?
- What can a suitable integrated MADM method be used to derive the criteria weight and ranking of alternatives?
- Are obtained results reliable and derived by the integrated MADM method?

This study has provided a systematic framework, which includes the BWM (best-worst method) method [19] integrated into the fuzzy-MARCOS (measurement of alternatives and ranking according to compromise solution) method [20] to select the best coating material for tooling industries. Evaluation and the rating of the coating alternatives and their criteria weight was done in the linguistic term given by the expert’s committee. As an approach toward real and practical scenarios, the alternatives were evaluated against the quantitative criteria. To deal effectively with these linguistic terms, it observed that fuzzy numbers are the best suitable number as the researchers wildly used them. Thus, we have to expand the MADM method to the fuzzy-MADM method. Here, coating material selection was considered as the case study. There are a total of eight coating materials (alternatives) that have been recognized. These coating alternatives are analyzed based on nine coating properties (criteria), which are selected in the presence of experts involved in coating application. We have used the above-mentioned fuzzy-MADM method to analyze and rank the alternatives. Finally, the obtained results were validated and tested by using the sensitivity analysis with four steps procedures. These procedures are (i) the effect of criteria weight change on the ranking, (ii) the effect of criteria weight derived from other methods, (iii) the effect of dynamic matrices on the ranking, and (iv) comparison with other well-established MADM methods. In addition to this, the proposed method is tested by solving the different published results for coating material selection.

The rest of the paper is organized in such a way that related work and research gaps are described in Section Related Work. A detailed description of the proposed methodology can be found in Section 2. Coating material selection is discussed in Section 3. The validation and testing of the proposed methodology have been elaborated in Section 4. The motive of the work combined with results and interpretation with a future scope is finally concluded in Section 5.

### Related Work

This section presents an overview of work done on the selection of coating materials for tooling industries. In the case of tooling industries, analyzing and selecting coating material is a serious issue. It is directly related to the failure of the die elements that interrupt continuous production, such as air-vent piercing and hole coining for steel wheels [6,21]. In this regard, countless studies are available on the selection of materials in various areas of application [22,23,24], but comparatively less work has been done on coating material selection. Refs [25,26,27] proposed a fuzzy TOPSIS approach to select a suitable coating material. In this approach, a Max-Min Set was used to determine the ordering value of the alternative, while TOPSIS was used to rank it. Sustainable coating material was selected to enhance the boiling heat transfer using multi-MADM methods, where the weight of criteria was calculated using entropy and AHP methods [28]. Suitable coating material for AISI4140 steel was selected using the TOPSIS method to improve the tribological properties of the steel, and the criteria weight was calculated using the entropy method [29]. A 316 SS coating material was selected as the best suitable material using the AHP-TOPSIS method to improve the corrosion resistance of magnesium alloy [30]. Robinson et al. [31] employed a PRECEPT knowledge-based computer system to select a suitable coating material to reduce the wear of surfaces and improve corrosion resistance. Firojkhan et al. [32] proposed a novel AHP-integrated TOPSIS approach to select a sustainable coating material for bearing application. They found that nitrided and WCC-coated steel was the most suitable material for the mentioned application. Chauhan et al. [33] used the TOPSIS method to rank the coating materials with the Ashby approach (Material selection chart). They concluded that TOPSIS and Ashby’s approach had good agreement with each other. Petković et al. [34] proposed the COPRAS and WASPAS methods, and the results were later compared with the TOPSIS method.

In addition to this, some researchers have used comparative experimental studies on coating material selection for tooling industries [16,17,35,36]. Kara et al. [37] deposited TiN, TiAlN, CrAlN, and TiAlN/TiSiN thin films using the cathodic arc evaporation method on AISI H13 tool steel (ø30 mm × 5 mm). The thicknesses of these coatings were 1.7 µm, 1.9 µm, 2.9 µm, and 2.3 µm, respectively. The frictional and wear behavior of coating was investigated against the Al_2_O_3_ counter body, and they found higher wear and scratch resistance for the CrAlN thin film along with nano hardness while lower wear and scratch resistance was observed for TAlN thin film. Souza et al. [38] deposited a thin film of AlCrN and TiAlN on AISI M2 tool steel. They found that AlCrN has good nanomechanical and wears resistance properties compared to TiAlN coating. Beake et al. [39] have conducted the micro-scratch test on TiAlN, AlCrN, and AlTiN coatings under different temperatures (25 °C and 500 °C). They observed that TiAlN coating has a lower critical load at 25 °C than AlCrN and AlTiN coating, whereas AlTiN shows excellent wear resistance compared to the AlCrN and TiAlN coatings. Dumkum et al. [40] deposited different coatings on tungsten carbide, and they observed that AlCrN/TiAlN coatings possess high hardness and TiAlN coating has low surface roughness, while AlCrN/TiN coatings have the lowest critical load (L_c1_) among all the coatings. AlCrN coating showed excellent wear resistance, while CrN coating revealed a low coefficient of friction [41]. Mechanical and tribological results show that AlCrN coating possesses high hardness and excellent wear resistance compared to the TiAlN and AlTiN coatings against boundary lubrication [42]. Chandrashekhar et al. [43] deposited AlCrN and TiAlN coatings on EN-353 steel, and they observed that AlCrN coating has excellent mechanical and wear properties compared to TiAlN coating.

The introductory literature on coating material selection proved that selection of the coating material is very tedious work. In past studies, researchers selected coating material based on a pilot experiment, which is also time-consuming and quite expensive. The experimental result interpretation and its comparison play a significant role in the selection of sustainable coating material. This process is only feasible for small numbers of coating materials. If the number of coating materials increases, it becomes more tedious and irritating to select the suitable coating material, and sometimes it leads to the wrong coating material selection. This inappropriate coating material increases the failure frequency of die elements, resulting in high maintenance time and machine ideal time. Thus, the present work proposes a fuzzy integrated MADM approach and its application to select sustainable coating material for tooling industries. In past studies [32,33], only bilayer coatings and mechanical properties were considered alternative and selection criteria. However, numerous coating materials (monolayer, bilayer, and multilayer) are available for tooling applications. The wear properties of coating material play a significant role in tooling application, which was not considered. Hence, in this study, the wear and mechanical properties of the coating material were considered as evaluation criteria.

## 2. The Proposed Integrated BWM Fuzzy-MADM Methodology

**Figure 1:**
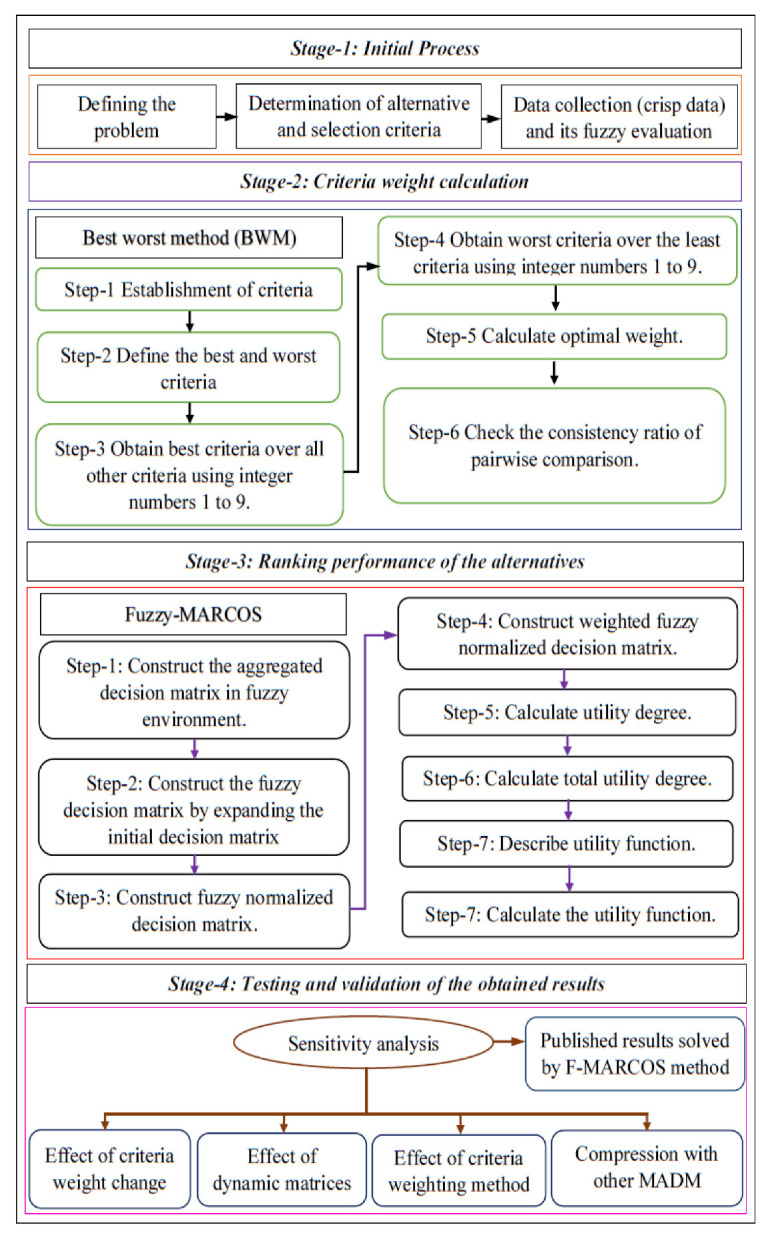
Algorithm of the proposed integrated BWM fuzzy MARCOS approach.

The proposed MADM method consists of four easy stages of the algorithm as presented in Figure 1. At the initial stage, a group of decision-makers was formed by the researcher, and the alternatives and their criteria weight were determined together.

In the next stage, the BWM method is implemented to calculate the criteria weight. At the same time, the fuzzy-MARCOS method is implemented to evaluate and rate the coating alternatives. Based on this evaluation, the alternative is ranked. Both methods are discussed briefly in Section 2.2.1 and Section 2.2.2, respectively. In the final stage, the obtained result is validated and tested using comprehensive sensitivity analysis and solving the other published results using the fuzzy-MARCOS method.

### 2.1. Fuzzy Membership Function

In this study, a particular type of triangular fuzzy number (TFN) was used [44], and it is presented as $\tilde{N}=(l,m,u)$ on ℝ. The membership function of it $\mu_{\tilde{N}}(x)$:$\mathbb{R}\to \left[0,1\right]$ is allocated by:

(1) $$\mu_{\tilde{N}}(x)=\left\{\begin{array}{c} \frac{x-l}{m-l}\;\;\;l\leq x\leq m\\ \frac{u-x}{u-m}\;\;\;m\leq x\leq u\\ \begin{array}{l} 0\;\;\;otherwise \end{array} \end{array}\right.$$

In the above expression, the lower and upper bounds of fuzzy number ($\tilde{N}$) are represented by *l* and *u*, respectively, while the model value is represented by m. The TFN is a commonly used fuzzy number that denotes the existence of imprecision in the expression. TFN and fuzzy set formulation are briefly presented in the following section. Many researchers have used TFN in the MADM method [45,46,47].

Following is the arithmetic operation for two triangular fuzzy numbers $\tilde{A}=\left(a^{l},a^{m},a^{u}\right)$ and $\tilde{B}=\left(b^{l},b^{m},b^{u}\right)$. [48]:

Addition:

(2) $$\tilde{A}+\tilde{B}=\left(a^{l}+b^{l},a^{m}+b^{m},a^{u}+b^{u}\right)$$

Subtraction:

(3) $$\tilde{A}-\tilde{B}=\left(a^{l}-b^{l},a^{m}-b^{m},a^{u}-b^{u}\right)$$

Multiplication:

(4) $$\tilde{A}\times \tilde{B}=\left(\min\left(a^{l}b^{l},a^{l}b^{u},a^{u}b^{l},a^{u}b^{u}\right),\max\left(a^{l}b^{l},a^{l}b^{u},a^{u}b^{l},a^{u}b^{u}\right)\right)$$

Division:

(5) $$\tilde{A}÷\tilde{B}=\left(\min\left(\frac{a^{l}}{b^{l}},\frac{a^{l}}{b^{u}},\frac{a^{u}}{b^{l}},\frac{a^{u}}{b^{u}}\right),\frac{a^{m}}{b^{m}},\max\left(\frac{a^{l}}{b^{l}},\frac{a^{l}}{b^{u}},\frac{a^{u}}{b^{l}},\frac{a^{u}}{b^{u}}\right)\right)$$

Scalar division: for positive real number *c*;

(6) $$\tilde{A}/c=\left(\frac{a^{l}}{c},\frac{a^{m}}{c},\frac{a^{c}}{c}\right)$$

### 2.2. Integrated Fuzzy MADM Method Comprising BWM and Fuzzy-MARCOS Approach

In this stage of the proposed methodology, the BWM method was implemented to derive the criteria weights. The method has a fundamental algorithm comprising six steps to implement. After deriving the criteria weight, fuzzy-MARCOS was implemented to determine the ranking of alternatives.

#### 2.2.1. BWM Method

The BWM method is the novel weight-choosing method to obtain the criteria weight and was developed by Professor Rezaei [19]. In this method, the decision-maker chooses the best and worst criteria representing a reference point for comparing the criteria. The reference comparison was carried out concerning the best and the worst criteria. This comparison was made between the best and other criteria; similarly, it was performed for the worst. This method also uses the idea of comparison between two criteria similar to the AHP method [49]. The BWM method requires a smaller comparison and is more consistent than the ANP, SMART, FARE, and AHP methods [50,51]. Finally, this method is well known for its simplicity because it compares criteria using an integer number between 1 and 9. The main advantage is that, unlike other methods, it does not use comparison matrices with fractional and integer numbers. Hence, the present study decided to use this method to determine the weight of the criteria and use it as additional proof of applicability. The basic algorithm of the BWM method is completed in the following six steps [19].

Step 1: Establish criteria set as $\left\{c_{1},c_{2},c_{3},\ldots \ldots c_{n}\right\}$.

Step 2: Obtain the best and worst criterion based on the assessment of the decision-maker.

Step 3: Obtain the most important criteria over all other criteria using integer numbers 1 to 9. The obtained BO (best to other) vector would be:

(7) $$A_{B}=(a_{B1},a_{B2},a_{B3},\ldots \ldots ,a_{Bn})$$

where the preference of the most important criteria (B) is denoted as aBj over the criterion j and aBB = 1.

Step 4: Obtain the preference of all the criteria over the least important criteria using integer numbers 1 to 9. The obtained OW (other to worst) vector would be:

(8) $$A_{W}={\left(a_{1W},a_{2W},a_{3W},\ldots \ldots ,a_{nW}\right)}^{T}$$

where the preference of criterion j is denoted by ajw over the least important criterion W and aww = 1.

Step 5: Calculate the optimal weight $\left(w_{1}^{*},w_{2}^{*},w_{3}^{*},\ldots \ldots ,w_{n}^{*}\right)$ The aim is to calculate the optimal weights for the criteria to minimize the maximum absolute differences for the wBwi−aBi and wiwW−aiW for all j. Based on the assumption (total sum of the weight equal to 1 and no negativity constraints), Rezaei [19] developed the linear BWM as follows:

(9) $$\begin{array}{c} \min\xi \\ s.\;t:\\ \left|w_{i}-a_{iw}w_{W}\right|\leq \xi ,\;\;\;i=1,2,3,\ldots \ldots ,n\;\\ \left|w_{B}-a_{Bi}w_{i}\right|\leq \xi ,\;\;\;i=1,2,3,\ldots \ldots ,n\;\\ \sum_{i=1}^{n}w_{i}=1\\ w_{i}\geq 0,\;\;\;j=1,2,3,\ldots \ldots ,n \end{array}$$

Step 6: Finally, the consistency ratio needs to be calculated using Equation (10), which checks the consistency of pairwise comparison. If the calculated value follows this equation means pairwise comparison possesses full consistency; otherwise, inconsistency occurs. The value of the consistency index (CI) is presented in [19].

(10) $$CR=\frac{\xi }{CI}$$

#### 2.2.2. Preference Evaluation of Coating Alternatives Using Fuzzy-MARCOS Approach

In order to perform the determination of significance values of the alternatives, the fuzzy-MARCOS method integrated with the BWM method has been executed. This method works based on a defined relationship between alternatives and their reference point (ideal and non-ideal alternatives). Further, the utility function needs to be calculated for each alternative using this defined alternative. In relation to ideal and non-ideal solutions, the compromised ranking was obtained. The value of the utility function denotes the actual location of the alternative concerning the ideal and non-ideal solutions. The most suitable alternative is nearer to the ideal solution and far from the non-ideal solution. The basic algorithm of this methodology comprises mainly ten steps [44]:

Here COm_1_, COm_2_, ……, and COm_n_ represent the alternatives, while the criteria are represented by CCO1, CCO2,……..., CCOn.

Step 1: Construct the aggregated decision matrix in a fuzzy environment. The panel of decision-makers constructed this matrix, and it is represented as

(11) $$\tilde{D}={\left[{\tilde{r}}_{ij}\right]}_{m\times n}$$

Here, ${\tilde{r}}_{ij}$ is the aggregated rating value of ith alternative with respect to jth criterion, and it is obtained using Equation (12)

(12) $${\tilde{r}}_{ij}={\prod_{k=1}^{K}\left({\tilde{r}}_{ijk}\right)}^{1/K},i=1,2,3\ldots \ldots ,m;j=1,2,3\ldots \ldots n$$

In the above expression, fuzzy preference rating is denoted by ${\tilde{r}}_{ijk}$, and it is also an element of a matrix presented in Equation (11). It is determined for *i*th alternative with respect to criterion jth by kth expert. This matrix is constructed using a geometric mean.

Step 2: Construct the fuzzy decision matrix by expanding the initial matrix (Equation (13)). In this step, the best (AI—Ideal solution) and worst (AAI—Anti Ideal solution) preference values of the options with respect to criterion are derived. The values are integrated into the expanded fuzzy decision matrix. Maximum value will consider beneficial criteria as the best preference, while minimum value will consider cost criteria as the worst preference. The lowest value will be the ideal solution, and the highest value will be the anti-ideal solution in the case of cost criteria. The expanded fuzzy decision matrix is constructed as follows:

(13) $$\tilde{D}=\begin{array}{c} \begin{array}{l} AAI\\ Cm_{1}\\ Cm_{2} \end{array}\\ \vdots \\ Cm_{m}\\ AI \end{array}\overset{\begin{array}{cccc} C_{1} & C_{2} & \cdots & C_{n} \end{array}}{\left[\begin{array}{cccc} \begin{array}{l} {\tilde{r}}_{aai1}\\ {\tilde{r}}_{11}\\ {\tilde{r}}_{21} \end{array} & \begin{array}{l} {\tilde{r}}_{aai2}\\ {\tilde{r}}_{12}\\ {\tilde{r}}_{22} \end{array} & \begin{array}{l} \cdots \\ \cdots \\ \cdots \end{array} & \begin{array}{l} {\tilde{r}}_{aain}\\ {\tilde{r}}_{1n}\\ {\tilde{r}}_{2n} \end{array}\\ \vdots & \vdots & \vdots & \vdots \\ {\tilde{r}}_{m1} & {\tilde{r}}_{m2} & \cdots & {\tilde{r}}_{mn}\\ {\tilde{r}}_{ai1} & {\tilde{r}}_{ai2} & \cdots & {\tilde{r}}_{ain} \end{array}\right]}$$

Step 3: In this step, fuzzy normalized matrix is constructed using Equations (14) and (15) for beneficial and cost criteria, respectively.

(14) $${\tilde{n}}_{ij}=\left(n_{ij}^{l},n_{ij}^{m},n_{ij}^{u}\right)=\left(\frac{r_{ij}^{l}}{r_{ai}^{u}},\frac{r_{ij}^{m}}{r_{ai}^{u}},\frac{r_{ij}^{u}}{r_{ai}^{u}}\right)$$

(15) $${\tilde{n}}_{ij}=\left(n_{ij}^{l},n_{ij}^{m},n_{ij}^{u}\right)=\left(\frac{r_{ai}^{l}}{r_{ij}^{u}},\frac{r_{ai}^{l}}{r_{ij}^{m}},\frac{r_{ai}^{l}}{r_{ij}^{l}}\right)$$

Step 4: Generate the weighted fuzzy normalized decision matrix by multiplying the criteria weight by the fuzzy normalized decision matrix.

(16) $${\tilde{\upsilon }}_{ij}=\left(\upsilon_{ij}^{l},\upsilon_{ij}^{m},\upsilon_{ij}^{u}\right)={\tilde{n}}_{ij}⊗w_{j}=\left(n_{ij}^{l}\times w_{j},n_{ij}^{m}\times w_{j},n_{ij}^{u}\times w_{j}\right)$$

Step-5: Determine the total weight value for each alternative using following Equation (17):

(17) $${\tilde{S}}_{i}=\sum_{j=1}^{n}{\tilde{\upsilon }}_{ij}$$

Here, ${\tilde{S}}_{i}$ it represents the sum of a weighted normalized matrix with respect to ith alternative. Similarly, determine the total weighted value for ideal $\left({\tilde{S}}_{ai}\right)$ and non-ideal $\left({\tilde{S}}_{aai}\right)$ solutions, respectively.

Step 6: Calculate the utility degree of each alternative using Equations (18) and (19).

(18) $${\tilde{K}}_{i}^{-}=\frac{{\tilde{S}}_{i}}{{\tilde{S}}_{aai}}=\left(\frac{s_{i}^{l}}{s_{aai}^{u}},\frac{s_{i}^{m}}{s_{aai}^{m}},\frac{s_{i}^{u}}{s_{aai}^{l}}\right)$$

(19) $${\tilde{K}}_{i}^{+}=\frac{{\tilde{S}}_{i}}{{\tilde{S}}_{ai}}=\left(\frac{s_{i}^{l}}{s_{ai}^{u}},\frac{s_{i}^{m}}{s_{ai}^{m}},\frac{s_{i}^{u}}{s_{ai}^{l}}\right)$$

Step 7: Obtain the total utility degree of each alternative using Equation (20).

(20) $${\tilde{t}}_{i}=\left(t_{i}^{l},t_{i}^{m},t_{i}^{u}\right)={\tilde{K}}_{i}^{-}⊕{\tilde{K}}_{i}^{+}=\left(k_{i}^{-l}+k_{i}^{+l},k_{i}^{-m}+k_{i}^{+m},k_{i}^{-u}+k_{i}^{+u}\right)$$

Further, new fuzzy number $\left(\tilde{d}\right)$ is calculated using Equation (21).

(21) $$\tilde{d}=\left(d^{l},d^{m},d^{u}\right)$$

Here, $d^{l}=\underset{i}{\max t_{i}^{l}},\underset{i}{d^{m}=\max t_{i}^{m}},\underset{i}{d^{u}=\max t_{i}^{u}}$ Now, perform defuzzification to obtain defuzzified number using Equation (22).

(22) $$df_{crisp}=\frac{d^{l}+\left(4\times d^{m}\right)+d^{u}}{6}$$

Step 8: In this step, utility function is described for ideal and anti-ideal solution using Equations (23) and (24), respectively.

(23) $$f\left({\tilde{K}}_{i}^{+}\right)=\frac{{\tilde{K}}_{i}^{-}}{df_{crisp}}=\left(\frac{k_{i}^{-l}}{df_{crisp}},\frac{k_{i}^{-m}}{df_{crisp}},\frac{k_{i}^{-u}}{df_{crisp}}\right)$$

(24) $$f\left({\tilde{K}}_{i}^{-}\right)=\frac{{\tilde{K}}_{i}^{+}}{df_{crisp}}=\left(\frac{k_{i}^{+l}}{df_{crisp}},\frac{k_{i}^{+m}}{df_{crisp}},\frac{k_{i}^{+u}}{df_{crisp}}\right)$$

Step 9: Using Equation (25), calculate the utility function for each alternative.

(25) $$f\left(K_{i}\right)=\frac{K_{i}^{+}+K_{i}^{-}}{1+\frac{1-f\left(K_{i}^{+}\right)}{f\left(K_{i}^{+}\right)}+\frac{1-f\left(K_{i}^{-}\right)}{f\left(K_{i}^{-}\right)}}$$

Here, $K_{i}^{-}$, $K_{i}^{+}$, $f\left(K_{i}^{-}\right)$ and $f\left(K_{i}^{+}\right)$ denotes the proportional defuzzified values.

Step 10: Based on the obtained utility function, determine the ranking of alternatives. The highest value of the utility function is derived as the best alternative.

## 3. Coating Material Selection Using Integrated BWM Fuzzy-MARCOS Approach

In this part of the study, we have considered the coating material selection problem for tooling industries. As already discussed, the selection of suitable coating for tooling application cannot be solely dependent upon its mechanical properties. Several other criteria must be considered in selecting coating material. To solve this MADM problem, we have implemented the integrated MADM methodology as discussed previously (Section 2). In this study, eight alternatives (Table 1) were considered and evaluated based on the nine criteria (Table 2). Then, the initial fuzzy decision matrix consisting of eight coating material alternatives and nine significant criteria is presented in Table 3. The criteria values were taken from literature for the coating TiAlN, AlCrN, Cr-(CrN/TiN), TiN, Cr-(CrN/TiAlN), TiCrN, AlCrN/TiAlN and Cr_2_O_3_/TiAlN [13,15,16,21,52,53,54]. The values of coefficient of thermal expansion for the coating materials were derived from literature [55,56,57]. In the second step, the model is generated for determining the criteria weight.

After considering the selection criteria, the criteria weights were calculated using the BWM method. These weights are determined based on the opinion of experts (Supplementary Table S1). In this study, five experts were selected from different backgrounds related to the coating material application. The first expert has expertise in materials, the second in thin film and tribology, the third in sheet metal forming (tool design and production), and the fourth one in tool design for sheet metal forming, and the fifth one in steel wheel production (sheet metal forming) and tool maintenance.

According to the methodology, the best and worst criteria were initially identified, and pairwise comparison was performed. The selection of the best and worst criteria was based on a questionnaire in which experts were involved. The final criteria weights and inconsistency rate are tabulated in Table 4, where the inconsistency rate is 0.048, which is nearer to zero (0). It can be extended to 5.23 (under consistency index Table 4). Hence, it can be said that the pairwise comparison is more consistent.

### Rank Evaluation of the Alternatives Using Fuzzy-MARCOS Approach

Before normalizing the initial fuzzy decision matrix, the ideal (*AI*) and anti-ideal solution (*AAI*) need to be calculated for each alternative corresponding to each criterion. *AI* was the highest value of each criterion, while the lowest value was the *AAI* concerning each alternative. Next, with the help of Equations (14) and (15) of the fuzzy-MARCOS method, the value of the initial fuzzy decision matrix was normalized (Supplementary Table S2). In the third step, the value of criteria weights was multiplied by the corresponding element of the fuzzy normalized matrix to obtain the weighted fuzzy normalized matrix (Supplementary Table S3). In the next step, the value of ${\tilde{S}}_{i}$ each alternative was determined using Equation (17). Similarly, the total weighted value for an ideal and anti-ideal solution was calculated respectively (Supplementary Table S3). The fourth step was dealing with the calculation of the utility degree of the alternative using Equations (18) and (19), respectively (Table 5).

New fuzzy numbers $\tilde{d}$ and defuzzified values $df_{crisp}$ were calculated to obtain the value of $f\left({\tilde{K}}_{i}^{-}\right)$ and $f\left({\tilde{K}}_{i}^{+}\right)$. Using Equations (23) and (24), the utility function value of each alternative was calculated (Table 6). The final utility function value of alternatives was calculated using Equation (25). Using these values, a ranking of alternatives was derived.

The evaluated results showed that alternative Cm_7_ is the best alternative coating material for tooling applications, while COm_1_ is the worst alternative (Supplementary Table S2). The order of the alternatives is COm_2_ < COm_1_ < COm_6_ < COm_4_ < COm_3_ < COm_8_ < COm_7_ < COm_5_ in ascending order of value of utility function.

## 4. Sensitivity Analysis

This section tested and validated the obtained result of the proposed integrated fuzzy-MADM method by performing the sensitivity analysis. In this analysis, four-step procedures have been followed, and these procedures are (i) effect of criteria weight change on the ranking, (ii) effect of criteria weight derived from other methods, (iii) effect of dynamic matrices on the ranking, and (iv) comparison with other well established MADM methods. Not only this, but the proposed integrated fuzzy-MADM method is tested by solving the different published results for coating material selection.

### 4.1. Effect of Criteria Weight Change on the Ranking of Alternatives

**Figure 2:**
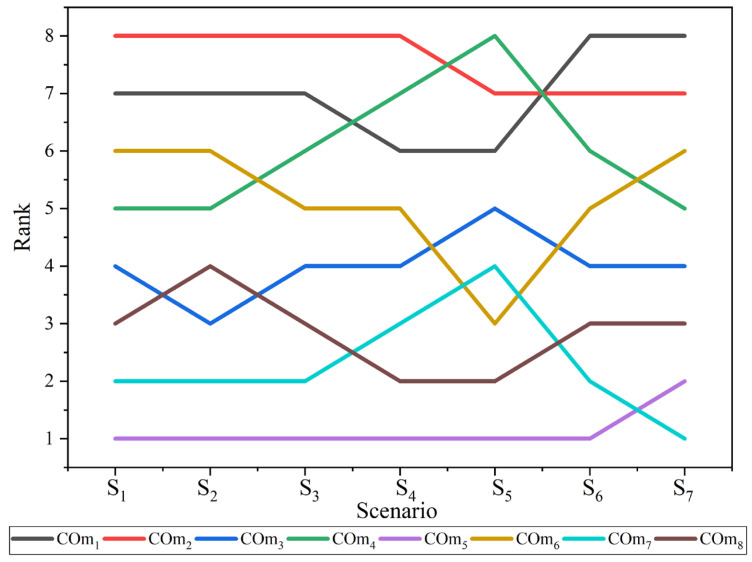
Ranking of alternatives for seven different scenarios.

In this analysis, a change in criteria weight was considered to observe how the ranking of alternatives reacts concerning weight change [58]. In the present study, seven different criteria weight scenarios were considered to perform this analysis (Table 7). The weights of the first five scenarios were determined using the BWM method by changing the preferences. In the sixth scenario, weight was equally distributed, while in the seventh scenario, half (0.5) of the criteria weight was assigned to criteria C_CO1_ and the remainder (0.0625) was equally distributed among other criteria. The ranking of the alternatives was derived using proposed MADM methods with these seven different criteria weights (Figure 2).

The obtained results showed that the ranking of alternatives changes with respect to the criteria weight change. It confirms that the proposed MADM methodology is sensitive to the criteria weight change. The evaluation of obtained results suggests that COm_5_ remains the best alternative for six scenarios, while it is the second-best alternative for the 7th scenario. This is sufficient enough to conclude that COm_5_ is the best coating material alternative. Additionally, COm_1_ is the worst alternative for the first four scenarios (S_1_, S_2_, S_3_, and S_4_), while it is the second worst alternative for the last three scenarios (S_5_, S_6_, and S_7_). The ranking obtained during the sensitivity test was also assisted by Spearman’s rank correlation coefficient between the original rank and ranking obtained from each scenario. The rank correlation coefficient values for each scenario are more than 0.91, and the mean value is 0.97. It shows a stronger correlation between ranks and credibility of the integrated BWM fuzzy-MARCOS method.

### 4.2. Effect of Dynamic Matrices on the Ranking of Alternatives

**Figure 3:**
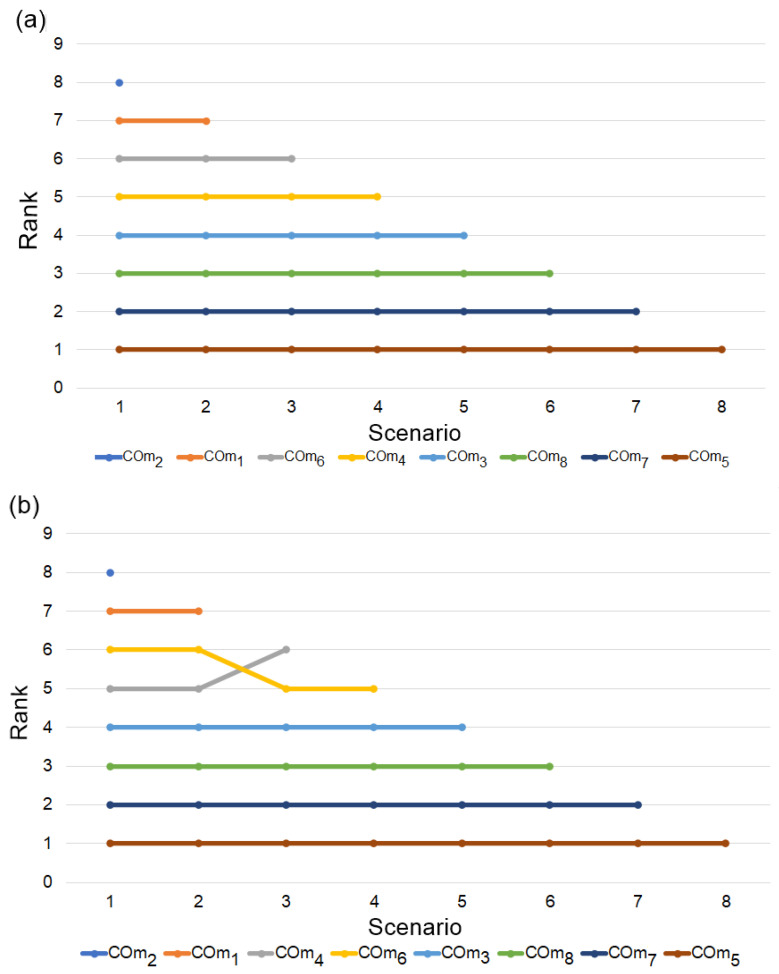
Effect of dynamic matrices on the ranking of alternatives derived using (**a**) fuzzy-MARCOS and (**b**) fuzzy-TOPSIS.

Substituting some parameters of the initial fuzzy decision matrix, such as removing the existing alternative or introducing a new alternative, can change the ranking of alternatives. In this section, several scenarios were formed by removing the existing initial fuzzy decision matrix alternative to simulate the performance. For each scenario, a new initial fuzzy decision matrix was created by removing the existing alternative, and then the proposed MADM method was applied. In this condition, the worst alternative of the existing matrix, i.e., COm_2_ (COm_2_ < COm_1_ < COm_6_ < COm_4_ < COm_3_ < COm_8_ < COm_7_ < COm_5_) was eliminated to create a new initial fuzzy decision matrix with seven alternatives. Then the new solution was obtained as follows: COm_1_ < COm_6_ < COm_4_ < COm_3_ < COm_8_ < COm_7_ < COm_5_. COm_1_ was identified as the worst alternative for the next scenario, and it was removed to create a new initial fuzzy decision matrix. Similarly, a total of seven scenarios were created, and the obtained result is presented in Figure 3a.

In the fuzzy-MARCOS algorithm, alternatives are examined based on ideal and anti-ideal reference points. The fuzzy-TOPSIS algorithm uses a similar concept to examine the alternatives [59]. So, the effect of dynamic matrices was performed by both methods (fuzzy-TOPSIS and fuzzy-MARCOS). Figure 3b shows that for scenarios S_3–_S_4_, the rank of the sixth alternative (Cm_6_) changes to the fifth position (rank reversal). At the same time, there is no such change observed for the fuzzy-MARCOS method when the initial fuzzy decision matrix was modified (Figure 3a). From both MADM methods, COm_5_ is the best alternative for all the scenarios. This indicates the stability and robustness of the integrated BWM fuzzy-MARCOS method in dynamic conditions.

### 4.3. Effect of Criteria Weights Derived by Different Weighting Methods

**Figure 4:**
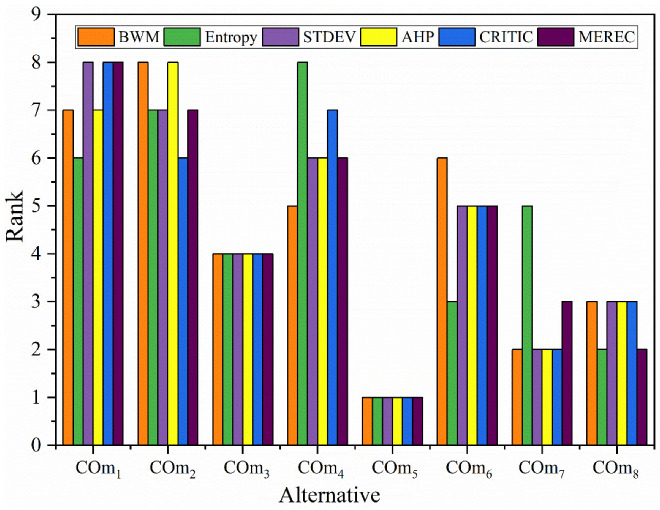
Effect of different criteria weighting methods on the ranking of alternative.

In this phase, criteria weights were derived using different weighing methods (Entropy [60], Standard deviation [61], AHP [62], CRITIC [63] and MEREC) [64]. Then, these weights were combined with fuzzy-MARCOS to determine the preference rating of the alternatives to compare the obtained results of the proposed BWM-integrated fuzzy-MADM method. The derived criteria weights are tabulated in Table 8, and the corresponding results are presented in Figure 4.

From comparative analysis (Figure 4), COm_5_ is observed as the best coating alternative from all the selected weighing methods integrated with the fuzzy-MARCOS method. It is similar to the ranking position obtained by the BWM-integrated fuzzy-MARCOS method. In addition to this, the ranking of alternative Cm_3_ does not change, while minor changes can be seen in the ranking of other alternatives, which did not affect the overall results. These changes in the ranking might be attributed to the different theoretical structures of weighing methods. The BWM method has a different procedure than the other weighing method, and it is based on linear programming [19], which helps the researcher to the quick generation of criteria weight. It is also a less time-consuming method to derive the criteria weight. In this perspective, BWM can contribute a more relevant and convenient algorithm than can be used by the researcher. Spearman’s rank correlation coefficient analyzed the correlation between the selected weighing methods and the proposed BWM method. The correlation coefficient between all the methods was found to be more than 0.93, except for the BWM and Entropy methods (0.65). These findings indicate that all the methods are strongly correlated with each other.

### 4.4. Comparison with other MADM Methods

**Figure 5:**
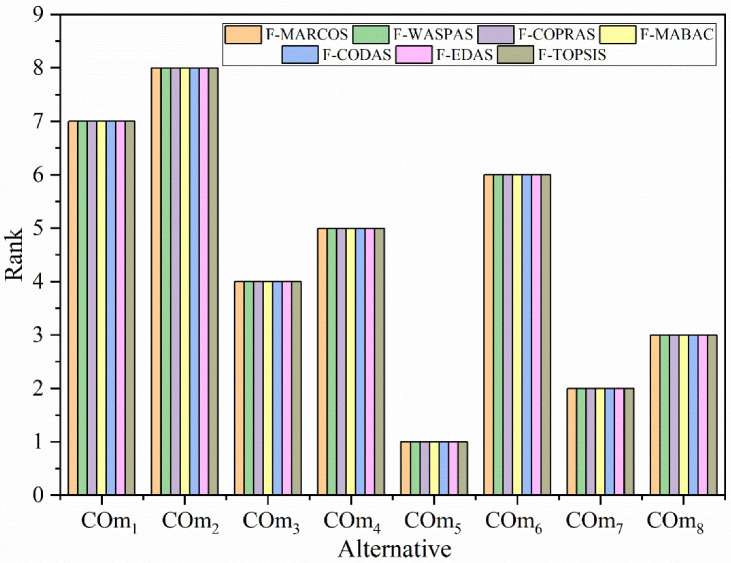
Ranking of alternative using different MADM methods.

In this phase of sensitivity analysis, six different integrated fuzzy-MADM methods were considered to check the result obtained by the fuzzy-MARCOS method. The considered MADM methods are fuzzy-WASPAS [65], fuzzy-COPRAS [66], fuzzy-MABAC [67], fuzzy-CODAS [68], fuzzy-EDAS [69], and fuzzy-TOPSIS [59]. During the evaluation of the performance rating of the alternative, the same criteria weights used for the fuzzy-MARCOS method were used, and the obtained results are presented in Figure 5.

The obtained results from different MADM methods show there is no difference in the ranking of the best alternative, COm_5_ (AlCrN/TiAlN) (Figure 5). There is also no change in the ranking of other alternatives except COm_5_. Alternative COm_5_ has allotted the eighth rank by fuzzy-TOPSIS method while other methods are given the seventh rank. This minor change does not affect the overall results of this study. Similar results obtained by all the MADM methods confirm the result obtained by the fuzzy-MARCOS method. Hence, this proved that the proposed integrated BWM fuzzy-MARCOS approach could be used to solve this type of problem.

### 4.5. Other Coating Material Selection Problems were Solved Using the Integrated BWM Fuzzy-MARCOS Approach

This section deals with the problem solving by the fuzzy-MARCOS method for other coating material selection, which past researchers have solved. This section has taken three different coating material selection problems that were solved using different MADM methods. The first example was for hard coating material selection. The second was to solve the coating material selection to enhance the heat transfer performance. The third was to solve a coating material selection problem for magnesium alloy.

#### 4.5.1. Hard Coating Material Selection

Chauhan and Vaish [33] have adopted the TOPSIS approach to solving the hard coating material selection problem. The other researcher also solved this coating material selection problem using COPRAS and WASPAS approaches [34]. Alternatives and selection criteria of the hard coating are presented in Table 9 and Table 10, respectively.

All the demonstrated criteria are beneficial criteria, except the thermal expansion coefficient, which is a non-beneficial criterion. The obtained criteria weights using BWM method are *w_C_*_1_ = 0.417, *w_C_*_2_ = 0.167, *w_C_*_3_ = 0.25, *w_C_*_4_ = 0.125, and *w_C_*_5_ = 0.042. The performance evaluation of the alternative was done using the fuzzy-MARCOS method (Section 2.2.2), and the results are presented in Table 9.

The obtained results (Table 11) noticed that Cm1 is the best alternative, while Cm37 is the worst coating alternative, and this ranking exactly matched with the ranking obtained by the past study. Further, Spearman’s rank correlation coefficient between the proposed approach and the existing approach was determined. The obtained average coefficient value (0.9) suggests that these approaches are strongly correlated. Hence, the obtained results are acceptable.

**Figure 6:**
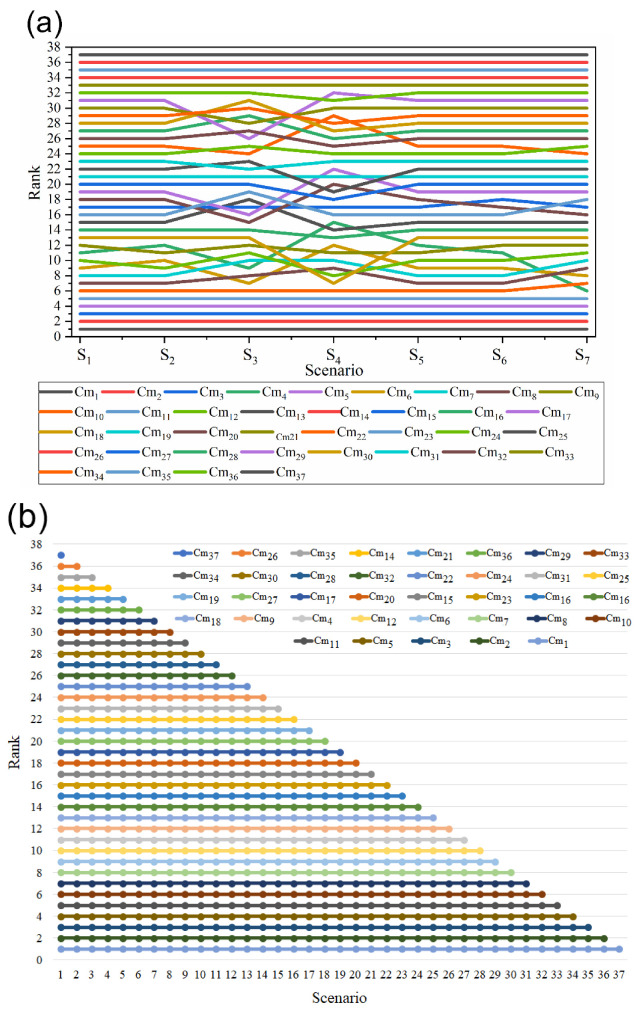

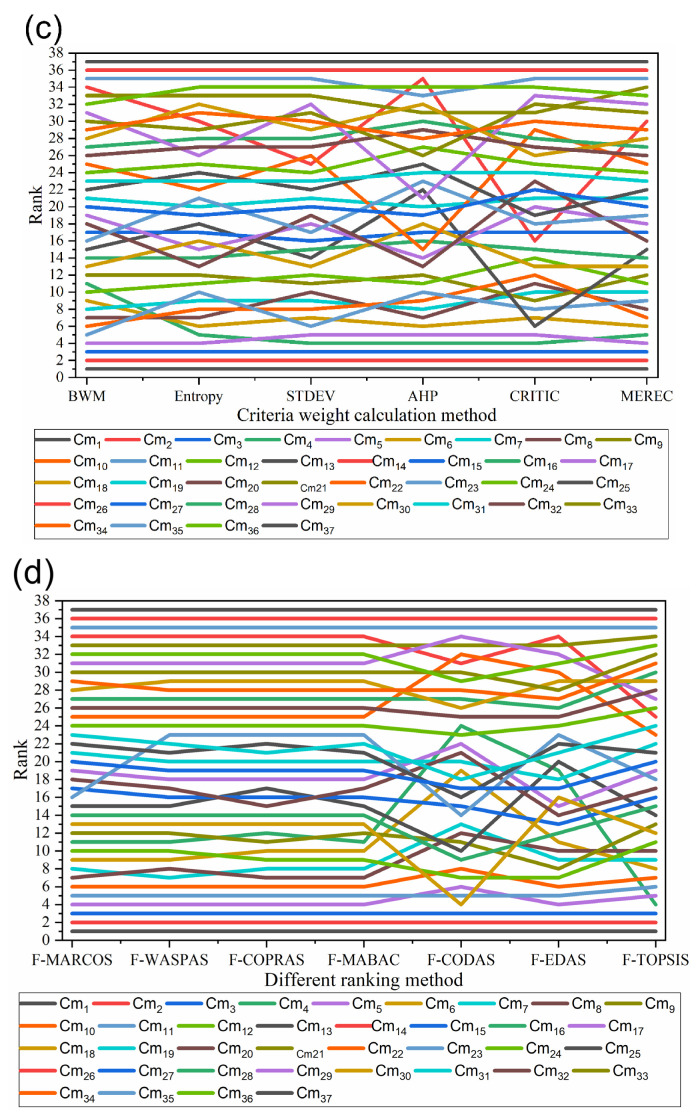
Results of sensitivity analysis (**a**) effect of criteria weight change, (**b**) effect of dynamic matrices, (**c**) effect of different criteria weight calculation method, and (**d**) comparison with other MADM methods.

Additionally, sensitivity analysis was performed to validate the obtained results, and this analysis has three phases, as is discussed in Section 4.1, Section 4.2, Section 4.3, and Section 4.4. Even though the ranking of some alternatives is changed according to the different scenarios, the best and worst alternative ranking does not change. It indicates the stability and reliability of the proposed BWM fuzzy-MARCOS method. The effect of a criteria weight change on the ranking is represented in Figure 6a, while the effect of the dynamic matrices is shown in Figure 6b. Figure 6c shows the effect of different criteria weight calculation methods on the ranking of alternatives, whereas Figure 6d represents the ranking of alternatives derived from BWM integrated different fuzzy-MADM methods. Furthermore, it was observed that the sensitivity analysis rankings have an excellent correlation between them, as the average Spearman’s rank correlation coefficient is 0.98.

#### 4.5.2. Coating Material Selection to Enhance the Heat Transfer Performance

In the past study, this coating material selection problem was solved using three different MADM methods (VIKOR, TOPSIS, and COPRAS) [28]. This coating material (see Table 12) was evaluated using four different criteria as shown in Table 13, it was taken from the past study [28]. Out of the four criteria, two are beneficial (thickness and thermal expansion coefficient), and two are non-beneficial criteria (cost and contact angle). The weights of these criteria, which were calculated using BWM methods and that, are *w_CHT_*_1_ = 0.068, *w_CHT_*_2_ = 0.632, *w_CHT_*_3_ = 0.181, and *w_CHT_*_4_ = 0.12. This problem was solved using the fuzzy-MARCOS method following the procedure mentioned in Section 2.2.2.

From Table 14, it is observed that the ranking of the best alternative is similar to the ranking obtained in the past study [28]. There are a majority of MADM methods, which suggests that CO_HT3_ (CNT coating) is the best alternative. The Spearman’s rank correlation coefficient between the ranking of alternatives was obtained in the acceptable range, i.e., > 0.8. It reveals that the rankings have an excellent correlation between them.

**Figure 7:**
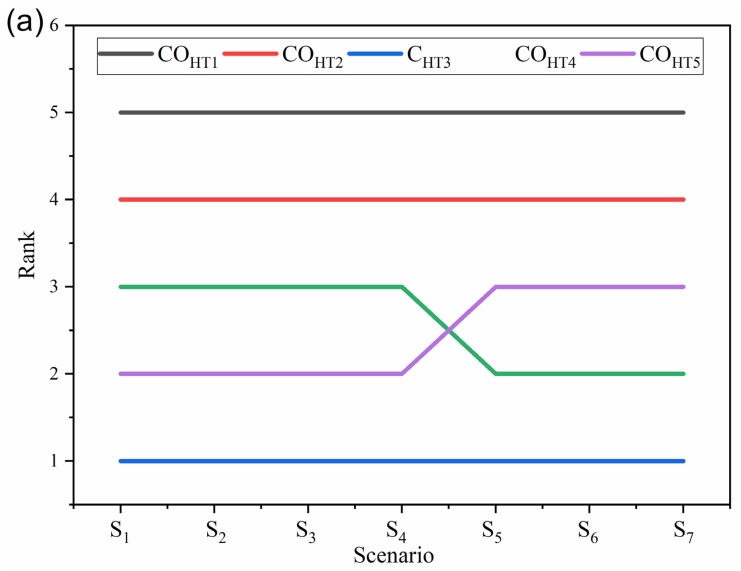

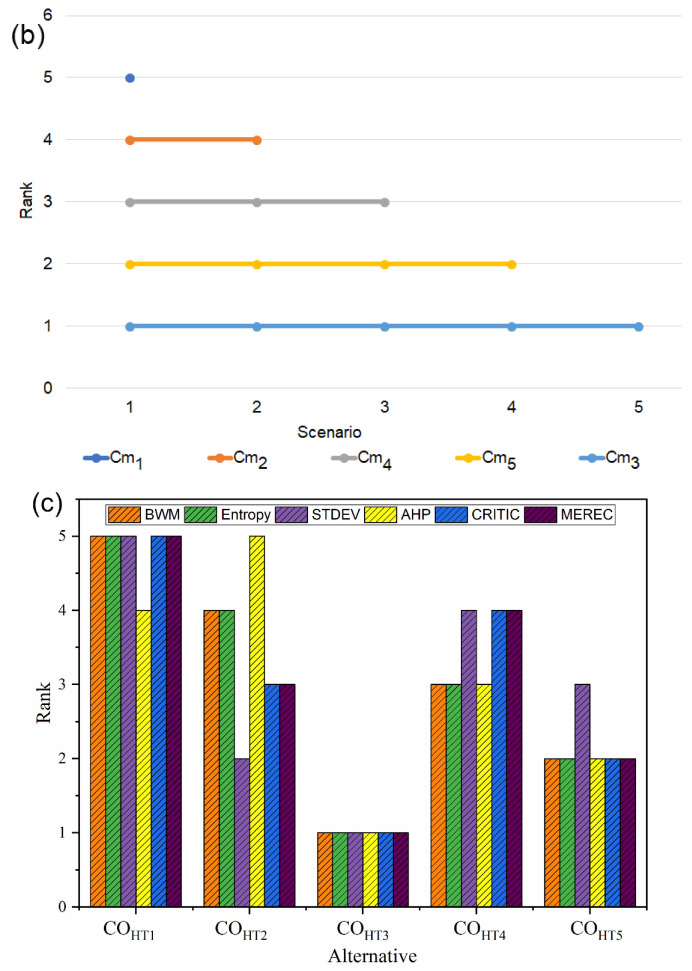

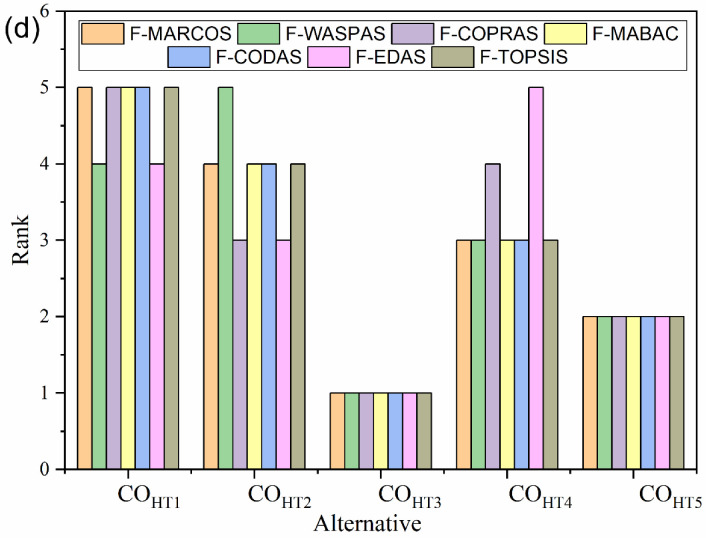
Consequences of (**a**) criteria weight change and (**b**) dynamic matrices, ranking of alternative derived from different (**c**) criteria weight change method and (**d**) other MADM approaches.

In addition to this, the obtained results were validated by performing the sensitivity analysis using four-phase methods (see Section 4.1, Section 4.2, Section 4.3, and Section 4.4). All four phases have the same opinion for the ranking of the best alternative (CO_HT3_-CNT coating). Figure 7 shows the effect of criteria weight change on the ranking of alternatives. The obtained ranking of alternatives has an excellent correlation as Spearman’s rank correlation coefficient is more than 0.9. Similarly, the effect of dynamic matrices is presented in Figure 7b, where no changes are observed in the ranking of alternatives. The ranking of the best and worst alternatives is similar even though the criteria weight calculation method was changed (Figure 7c), and the correlation coefficient between these rankings is in the acceptable range (>0.7). A similar coefficient value (>0.7) is obtained when the ranking was obtained using different fuzzy-MADAM methods. In this phase of sensitivity analysis, the ranking of the best alternative does not change, whereas the ranking of the worst alternative changes in the fuzzy-WASPAS and fuzzy-EDAS methods (Figure 7d).

#### 4.5.3. Coating Material Selection for Magnesium Alloy

The present coating material selection problem was solved in past research by Prasad et al. [30] using the fuzzy-AHP integrated TOPSIS method. The coating alternative and its evaluation criteria are presented in Table 15 and Table 16, respectively. There are three beneficial (quantitative, qualitative, and quality) and three non-beneficial (coating structure, cost, and risk factor) criteria identified. The weights of these criteria were derived using the BWM method and those are *w_CMA_*_1_ = 0.041, *w_CMA_*_2_ = 0.088, *w_CMA_*_3_ = 0.146, *w_CMA_*_4_ = 0.362, *w_CMA_*_5_ = 0.146, and *w_CMA_*_6_ = 0.219. This coating material selection problem was solved using the integrated BWM fuzzy-MARCOS method, and the results are presented in Table 11.

The obtained ranking by the fuzzy-MARCOS method remains similar to the ranking derived in the past study (Table 17). The present solution also suggests CO_MA1_ and CO_MA2_ are the best and worst alternatives for coating materials, respectively, for magnesium alloy. Both the rankings are strongly correlated with each other, as Spearman’s rank correlation coefficient is 0.82. Hence, the proposed method is an efficient and robust MADM tool to solve this type of problem.

**Figure 8:**
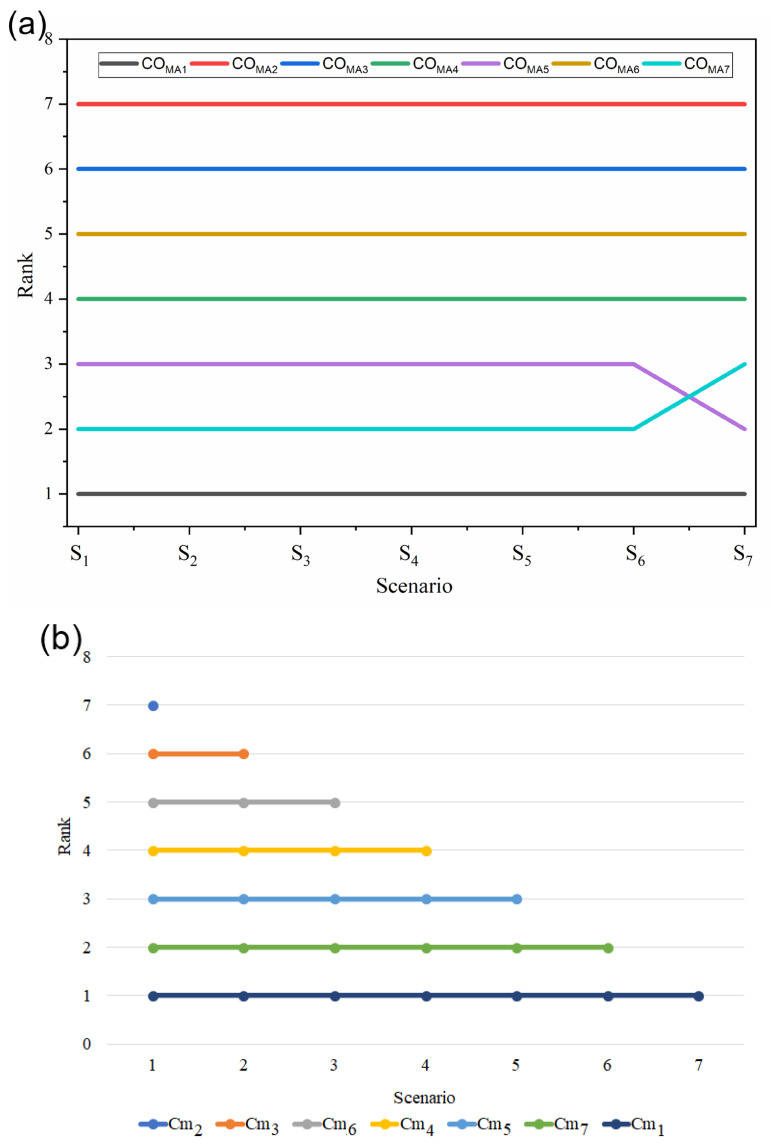

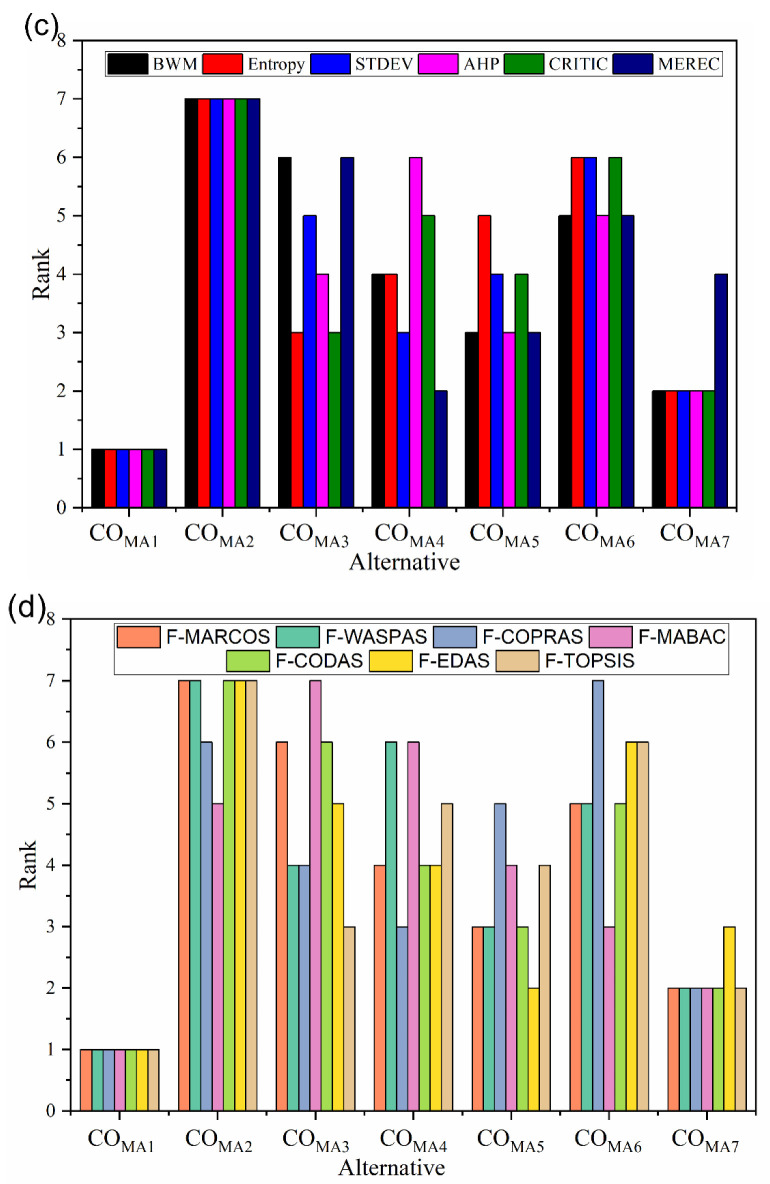
Results of sensitivity analysis, (**a**) effect of change in criteria weight and (**b**) dynamic matrices, (**c**) effect of different methods used for criteria weight calculation, and (**d**) ranking of alternative.

Furthermore, to analyze the validity of obtained ranking, four phases of comparative sensitivity analysis were conducted. These phases are elaborated in Section 4.1, Section 4.2, Section 4.3, and Section 4.4. There are few changes observed in the ranking of alternatives corresponding to the different phases of sensitivity analysis (Figure 8). But there are no such changes observed in the ranking of the best alternative, and it is similar for all the phases. Additionally, the correlation between rankings was derived using Spearman’s rank correlation coefficient, and the overall coefficient value is 0.89. It indicates that the rankings are excellently correlated with each other.

From the above discussion, the ranking of the best alternative COm_5_ (AlCrN/TiAlN) in the present study does not change, even though four phases of sensitivity analysis were used. Similarly, ranking the best alternative for other coating material sections also has the same result. This indicates the reliability and robustness of the proposed integrated BWM fuzzy-MARCOS approach.

## 5. Conclusions

This study proposes a convenient integrated fuzzy-MADM method comprising BWM and the fuzzy-MARCOS approach to solve the coating material selection problem. BWM and fuzzy-MARCOS are novel MADM methods that can be applied to solve other multi-attributed decision-making problems observed in the different fields. This study is the first to propose and utilize this approach to solve the coating material selection problem for tooling industries. The findings of this work proved that the proposed integrated model was effectively executed, and it gives valuable knowledge regarding the coating material section for the tooling industries.

The alternative Cm5 (AlCrN/TiAlN) was selected as the most suitable coating material among eight coating material alternatives for tooling application. This coating material has an excellent combination of mechanical properties (*H_IT_* = 38 GPa, *E_IT_* = 423 GPa, *H_IT_/E_IT_* = 0.09, $H_{IT}^{3}$*/*$E_{IT}^{2}$ = 0.313 GPa, *n* = 0.44,) and wear (*Ra* = 0.24 µm, *CoF* = 0.49, *WML* = 6.61 mg). The analytical findings of this study have a critical suggestion for the tool designer and the experts of coating materials regarding the applicability of the proposed method which can be efficiently used for material selection. Additionally, the proposed MADM method can be modified and integrated with some other models which require further investigation. As this novel method has a wide application in the field of decision making and various components with different features can be compared by using this method to get the best one among the alternatives available for a specific study.

## Figures and Tables

**Table 1:** Coating material alternatives and their chemical composition.

Coating Materials Alternative	Symbol	Composition (at. %)				
		Ti	N	Cr	Al	O
TiN	COm_1_	38.63	61.37	-	-	-
TiCrN	COm_2_	27.97	51.21	20.82	-	-
TiAlN	COm_3_	26.83	48.08	-	25.14	-
AlCrN	COm_4_	-	47.82	19.99	32.19	-
AlCrN/TiAlN	COm_5_	20.31	56.11	2.32	20.66	-
Cr-(CrN/TiN)	COm_6_	36.96	58.73	4.31	-	-
Cr-(CrN/TiAlN)	COm_7_	22.53	56.18	3.5	17.79	-
Cr_2_O_3_/TiAlN	COm_8_	24.33	48.52	5.57	16.76	4.82

Each coating has similar thickness i.e., ~ 3 ± 0.2 μm.

**Table 2:** Selection criteria of coating material.

Properties of Coating Materials (Criteria)	Symbol
Indentation hardness (H_IT_, in Gpa)	C_CO1_
Young’s modulus (E_IT_, in GPa)	C_CO2_
Wear resistance (H_IT_/E_IT_)	C_CO3_
Plastic Deformation ((H_IT_)^3^/(E_IT_)^2^, in GPa)	C_CO4_
Strain hardening exponent (n)	C_CO5_
Coefficient of thermal expansion (K^−1^, ×10^−6^)	C_CO6_
Surface roughness (Ra, in µm)	C_CO7_
Coefficient of friction (CoF)	C_CO8_
Wear rate (K, in mm^3^/Nm)	C_CO9_

**Table 3:** Initial fuzzy diction matrix for evaluating the coating material alternatives.

Alternative	C_CO1_	C_CO2_	C_CO3_	C_CO4_	C_CO5_
COm_1_	(18, 21, 24)	(362, 372, 382)	(0.050, 0.057, 0.063)	(0.045, 0.067, 0.095)	(0.31, 0.32, 0.33)
COm_2_	(17, 20, 23)	(318, 330, 342)	(0.053, 0.061, 0.067)	(0.049, 0.074, 0.104)	(0.29, 0.3, 0.31)
COm_3_	(31, 35, 39)	(350, 359, 368)	(0.089, 0.098, 0.106)	(0.243, 0.333, 0.438)	(0.39, 0.4, 0.41)
COm_4_	(27, 32, 37)	(361, 368, 375)	(0.075, 0.087, 0.099)	(0.151, 0.242, 0.360)	(0.33, 0.35, 0.37)
COm_5_	(35, 38, 41)	(409, 423, 437)	(0.086, 0.090, 0.094)	(0.256, 0.313, 0.361)	(0.43, 0.44, 0.45)
COm_6_	(26, 28, 30)	(339, 347, 355)	(0.077, 0.081, 0.085)	(0.153, 0.182, 0.214)	(0.32, 0.34, 0.36)
COm_7_	(37, 40, 43)	(358, 367, 376)	(0.103, 0.109, 0.114)	(0.395, 0.475, 0.562)	(0.41, 0.42, 0.43)
COm_8_	(33, 38, 43)	(374, 387, 400)	(0.088, 0.098, 0.108)	(0.257, 0.366, 0.497)	(0.35, 0.36, 0.37)
Alternative	C_CO6_		C_CO7_	C_CO8_	C_CO9_
COm_1_	(8.14, 9.35, 10.56)		(0.24, 0.26, 0.28)	(0.3, 0.32, 0.34)	(11.72, 12.46, 13.2)
COm_2_	(3.03, 3.21, 3.39)		(0.28, 0.31, 0.34)	(0.42, 0.43, 0.44)	(9.24, 9.73, 10.22)
COm_3_	(6.6, 7.5, 8.4)		(0.28, 0.32, 0.36)	(0.52, 0.55, 0.58)	(6.11, 6.62, 7.13)
COm_4_	(5.2, 5.9, 6.6)		(0.37, 0.39, 0.41)	(0.55, 0.57, 0.59)	(7.24, 7.87, 8.5)
COm_5_	(6.6, 7.5, 8.4)		(0.21, 0.24, 0.27)	(0.48, 0.49, 0.5)	(6.2, 6.61, 7.02)
COm_6_	(8.14, 9.35, 10.56)		(0.22, 0.27, 0.32)	(0.3, 0.33, 0.36)	(8.41, 8.94, 9.47)
COm_7_	(6.6, 7.5, 8.4)		(0.39, 0.42, 0.45)	(0.46, 0.48, 0.5)	(6.1, 6.49, 6.88)
COm_8_	(6.6, 7.5, 8.4)		(0.28, 0.31, 0.34)	(0.4, 0.43, 0.46)	(7.43, 7.9, 8.37)

**Table 4:** Weight of criteria and inconsistency rate (Ksi *).

Criteria	C_CO1_	C_CO2_	C_CO3_	C_CO4_	C_CO5_	C_CO6_	C_CO7_	C_CO8_	C_CO9_
Weight	0.254	0.151	0.076	0.061	0.152	0.027	0.1	0.076	0.103
Ksi *	0.048								

**Table 5:** Calculating and summarizing the utility degree and fuzzy matrix of ${\tilde{t}}_{i}$.

**Alternative**	${\tilde{S}}_{i}$	${\tilde{K}}_{i}^{-}$	${\tilde{K}}_{i}^{+}$	${\tilde{t}}_{i}$
*AAI*	(0.487, 0.528, 0.570)			
COm_1_	(0.571, 0.617, 0.664)	(1.003, 1.169, 1.365)	(0.571, 0.659, 0.758)	(1.574, 1.828, 2.123)
COm_2_	(0.548, 0.593, 0.639)	(0.962, 1.123, 1.313)	(0.548, 0.633, 0.729)	(1.511, 1.757, 2.041)
COm_3_	(0.717, 0.780, 0.848)	(1.258, 1.478, 1.742)	(0.717, 0.833, 0.967)	(1.974, 2.311, 2.709)
COm_4_	(0.638, 0.706, 0.778)	(1.120, 1.338, 1.599)	(0.638, 0.754, 0.888)	(1.758, 2.092, 2.487)
COm_5_	(0.801, 0.853, 0.908)	(1.405, 1.616, 1.865)	(0.801, 0.911, 1.035)	(2.206, 2.527, 2.900)
COm_6_	(0.649, 0.700, 0.757)	(1.140, 1.325, 1.555)	(0.649, 0.747, 0.863)	(1.789, 2.073, 2.419)
COm_7_	(0.786, 0.834, 0.885)	(1.379, 1.580, 1.818)	(0.786, 0.891, 1.009)	(2.165, 2.471, 2.827)
COm_8_	(0.725, 0.796, 0.871)	(1.273, 1.508, 1.790)	(0.725, 0.850, 0.994)	(1.998, 2.358, 2.784)
*AI*	(0.877, 0.936, 1.000)			$df_{crisp}$= 2.536

**Table 6:** Values of utility functions and final ranking of the alternatives.

Alternative	$f\left({\tilde{K}}_{i}^{-}\right)$	$f\left({\tilde{K}}_{i}^{+}\right)$	$K_{i}^{-}$	$K_{i}^{+}$	$f\left(K_{i}^{-}\right)$	$f\left(K_{i}^{+}\right)$	$f\left(K_{i}\right)$	Rank
COm_1_	(0.225, 0.260, 0.299)	(0.395, 0.461, 0.538)	1.174	0.661	0.261	0.463	0.145	7
COm_2_	(0.216, 0.250, 0.287)	(0.379, 0.443, 0.518)	1.128	0.635	0.250	0.445	0.133	8
COm_3_	(0.283, 0.329, 0.381)	(0.496, 0.583, 0.687)	1.485	0.836	0.330	0.586	0.245	4
COm_4_	(0.252, 0.297, 0.350)	(0.442, 0.527, 0.631)	1.345	0.757	0.299	0.530	0.196	5
COm_5_	(0.316, 0.359, 0.408)	(0.554, 0.637, 0.735)	1.622	0.913	0.360	0.640	0.299	1
COm_6_	(0.256, 0.295, 0.340)	(0.449, 0.523, 0.613)	1.333	0.750	0.296	0.526	0.192	6
COm_7_	(0.310, 0.351, 0.398)	(0.544, 0.623, 0.717)	1.586	0.893	0.352	0.626	0.284	2
COm_8_	(0.286, 0.335, 0.392)	(0.502, 0.595, 0.706)	1.516	0.853	0.336	0.598	0.256	3

**Table 7:** Different scenarios of criteria weightage for sensitivity analysis.

Scenarios	Criteria Weightage								
	C_CO1_	C_CO2_	C_CO3_	C_CO4_	C_CO5_	C_CO6_	C_CO7_	C_CO8_	C_CO9_
Scenario 1 (S_1_)	0.254	0.151	0.076	0.061	0.152	0.027	0.1	0.076	0.103
Scenario 2 (S_2_)	0.149	0.138	0.078	0.066	0.284	0.020	0.062	0.080	0.123
Scenario 3 (S_3_)	0.110	0.039	0.055	0.190	0.094	0.068	0.300	0.047	0.097
Scenario 4 (S_4_)	0.110	0.018	0.087	0.190	0.019	0.038	0.400	0.078	0.060
Scenario 5 (S_5_)	0.016	0.018	0.099	0.190	0.019	0.038	0.400	0.160	0.060
Scenario 6 (S_6_)	0.111	0.111	0.111	0.111	0.111	0.111	0.111	0.111	0.111
Scenario 7 (S_7_)	0.500	0.063	0.063	0.063	0.063	0.063	0.063	0.063	0.063

**Table 8:** Criteria weights derived by the different weighing methods.

Weighing Methods	C_CO1_	C_CO2_	C_CO3_	C_CO4_	C_CO5_	C_CO6_	C_CO7_	C_CO8_	C_CO9_
Entropy	0.091	0.008	0.068	0.119	0.026	0.055	0.494	0.060	0.080
STDEV	0.125	0.113	0.112	0.113	0.113	0.085	0.112	0.118	0.109
AHP	0.241	0.092	0.081	0.141	0.125	0.035	0.173	0.064	0.048
CRITIC	0.089	0.082	0.087	0.085	0.082	0.148	0.154	0.191	0.084
MEREC	0.115	0.030	0.103	0.089	0.052	0.090	0.325	0.078	0.117

**Table 9:** Hard coating materials alternative.

Coating Materials Alternatives	Symbol	Coating Materials Alternatives	Symbol	Coating Materials Alternatives	Symbol
C	Cm_1_	CrN	Cm_14_	HfO_2_	Cm_26_
BN	Cm_2_	ZrB_2_	Cm_15_	CrB_2_	Cm_27_
B_4_C	Cm_3_	NbB_2_	Cm_16_	ZrN	Cm_28_
Si_3_N_4_	Cm_4_	Al_2_O_3_	Cm_17_	TiO_2_	Cm_29_
VC	Cm_5_	W_2_B_5_	Cm_18_	TaC	Cm_30_
SiB_6_	Cm_6_	VB_2_	Cm_19_	TiN	Cm_31_
LaB_6_	Cm_7_	Cr_3_C_2_	Cm_20_	Mo_2_C	Cm_32_
ZrC	Cm_8_	AlN	Cm_21_	BeO	Cm_33_
SiC	Cm_9_	ZrO_2_	Cm_22_	VN	Cm_34_
TiC	Cm_10_	Mo_2_B_5_	Cm_23_	ThO_2_	Cm_35_
TiB_2_	Cm_11_	NbC	Cm_24_	NbN	Cm_36_
B	Cm_12_	TaB_2_	Cm_25_	MgO	Cm_37_
WC	Cm_13_				

**Table 10:** Selection criteria of hard coating material.

Properties of Coating Materials (Criteria)	Symbol
Hardness (H_IT_, in Gpa)	C_1_
Young’s modulus (E_IT_, in GPa)	C_2_
Wear resistance (H_IT_/E_IT_)	C_3_
Plastic Deformation ((H_IT_)^3^/(E_IT_)^2^, in GPa)	C_4_
Strain hardening exponent (n)	C_5_

**Table 11:** The final preference value of alternative and its ranking.

Alternative	*f(K_i_)*	F-MARCOS	TOPSIS [33]	COPRAS [34]	WASPAS [34]	Alternative	*f(K_i_)*	F-MARCOS	TOPSIS [33]	COPRAS [34]	WASPAS [34]
Cm_1_	0.123	1	1	1	1	Cm_20_	0.014	18	20	19	19
Cm_2_	0.056	2	2	2	2	Cm_21_	0.006	33	21	32	33
Cm_3_	0.034	3	3	3	3	Cm_22_	0.010	25	22	26	28
Cm_4_	0.018	11	4	4	5	Cm_23_	0.014	16	23	20	18
Cm_5_	0.024	4	5	5	4	Cm_24_	0.010	24	24	25	24
Cm_6_	0.019	9	6	6	7	Cm_25_	0.012	22	25	23	22
Cm_7_	0.020	8	7	7	9	Cm_26_	0.004	36	26	36	36
Cm_8_	0.020	7	8	8	10	Cm_27_	0.014	20	27	22	21
Cm_9_	0.018	12	9	11	11	Cm_28_	0.009	27	28	27	27
Cm_10_	0.021	6	10	10	8	Cm_29_	0.007	31	29	33	32
Cm_11_	0.022	5	11	9	6	Cm_30_	0.008	28	30	29	29
Cm_12_	0.019	10	12	12	12	Cm_31_	0.012	23	31	24	23
Cm_13_	0.015	15	13	13	13	Cm_32_	0.009	26	32	28	26
Cm_14_	0.006	34	14	18	25	Cm_33_	0.008	30	33	31	30
Cm_15_	0.014	17	15	16	16	Cm_34_	0.008	29	34	30	31
Cm_16_	0.017	14	16	15	15	Cm_35_	0.005	35	35	35	35
Cm_17_	0.014	19	17	17	17	Cm_36_	0.007	32	36	34	34
Cm_18_	0.018	13	18	14	14	Cm_37_	0.003	37	37	37	37
Cm_19_	0.013	21	19	21	20						

**Table 12:** Alternatives for coating materials.

Coating Materials Alternatives	Symbol
Al_2_O_3_	CO_HT1_
TiO_2_	CO_HT2_
CNT	CO_HT3_
SiO_2_	CO_HT4_
ZnO	CO_HT5_

**Table 13:** Evaluation criteria of coating materials.

Properties of Hard Coating (Criteria)	Symbol
Cost	C_HT1_
Contact angle	C_HT2_
Coefficient of thermal expansion (K^−1^, ×10^−6^)	C_HT3_
Thickness	C_HT4_

**Table 14:** Final ranking of the alternatives.

Alternative	*f(K_i_)*	F- MARCOS	AHP-VIKOR [28]	E-VIKOR [28]	AHP-COPRAS [28]	E-COPRAS [28]	AHP-TOPSIS [28]	E-TOPSIS [28]
CO_HT1_	0.042	5	4	4	4	4	2	2
CO_HT2_	0.051	4	5	2	5	5	1	3
CO_HT3_	0.169	1	1	5	1	1	5	1
CO_HT4_	0.096	3	3	1	2	3	4	4
CO_HT5_	0.124	2	2	3	3	2	3	5

**Table 15:** Alternatives of coating materials for magnesium alloy.

Coating Materials Alternatives	Symbol
316SS	CO_MA1_
Al_2_O_3_-TiO_2_	CO_MA2_
Zn/Al-Mn Composite	CO_MA3_
Si3N4	CO_MA4_
NiCrBSi	CO_MA5_
CoNiCrAlY	CO_MA6_
Ni-Zn-Cu-P/Ni-P duplex	CO_MA7_

**Table 16:** Evaluation criteria of coating materials.

Properties of Hard Coating (Criteria)	Symbol
Quantitative	C_MA1_
Qualitative	C_MA2_
Cost	C_MA3_
Quality	C_MA4_
Coating structure	C_MA5_
Risk factor	C_MA6_

**Table 17:** Final preference value and ranking of the coating alternative.

Alternative	*f(K_i_)*	F-MARCOS	TOPSIS [30]
CO_MA1_	0.308	1	1
CO_MA2_	0.227	7	7
CO_MA3_	0.230	6	4
CO_MA4_	0.245	4	3
CO_MA5_	0.249	3	5
CO_MA6_	0.237	5	6
CO_MA7_	0.304	2	2

## Data Availability

All the data has been incorporated into the manuscript.

## Supplementary Materials

The following supporting information can be downloaded at: https://www.mdpi.com/article/10.3390/ma15249002/s1. Table S1 Pairwise comparison vector for best to other criteria and other to worst criteria. Table S2 Normalized fuzzy decision matrix. Table S3 Weighted normalized fuzzy decision matrix.

## Abbreviations

MADM	Multi attributed decision making
BWM	Best Worst method
AHP	Analytic Hierarchy Process
STDEV	Standard deviation
CRITIC	Criteria Importance Through Intercriteria Correlation
MEREC	Method Based on the Removal Effects of Criteria
F-MARCOS	Fuzzy Measurement Alternatives and Ranking according to the Compromise Solution
F-TOPSIS	Fuzzy Technique for Order of Preference by Similarity to Ideal Solution
F-COPRAS	Fuzzy Complex Proportional Assessment
F-WASPAS	Fuzzy Weighted Aggregates Sum Product Assessment
F-MABAC	Fuzzy multi-attributive border approximation area comparison
F-CODAS	Fuzzy Combinative Distance-based Assessment
F-EDAS	Fuzzy Evaluation Based on Distance from Average Solution
