# Supplementary material for: Application of Integrated BWM Fuzzy-MARCOS Approach for Coating Material Selection in Tooling Industries
Source: Materials (Basel). 2022 Dec 16;15(24):9002. doi: 10.3390/ma15249002 (PMC9782703; doi:10.3390/ma15249002)
Supplement: Supplementary file 1 [file materials-15-09002-s001.zip › materials-2038883-supplementary.pdf]

Supporting data:

Supplementary Table S1 Pairwise comparison vector for best to other criteria and other to worst criteria

Best to others criteria	*H*	*E*	*H/E*	H^3/E^2	*n*	*Ra*	*α*	*CoF*	*WML*
Best criteria: *Hardness (H)*	1	2	4	5	2	3	8	4	2
Other to worst criteria	Worst criteria: *Coefficient of thermal expansion (α)*								
H	8								
E	6								
H/E	3								
H^3/E^2	4								
n	6								
Ra	5								
α	1								
CoF	3								
WML	2								

Supplementary Table S2 Normalized fuzzy decision matrix

Alternative	C_1	C_2	C_3	C_4	C_5
AAI	(0.395, 0.465, 0.535)	(0.728, 0.755, 0.783)	(0.435, 0.498, 0.549)	(0.079, 0.119, 0.168)	(0.644, 0.667, 0.689)
Cm_1	(0.419, 0.488, 0.558)	(0.828, 0.851, 0.874)	(0.435, 0.498, 0.549)	(0.079, 0.119, 0.168)	(0.689, 0.711, 0.733)
Cm_2	(0.395, 0.465, 0.535)	(0.728, 0.755,0.783	(0.467, 0.533, 0.588)	(0.086, 0.132, 0.185)	(0.644, 0.667, 0.689)
Cm_3	(0.721, 0.814, 0.907)	(0.801, 0.822, 0.842)	(0.774, 0.857, 0.927)	(0.432, 0.592, 0.779)	(0.867, 0.889, 0.911)
Cm_4	(0.628, 0.744, 0.860)	(0.826, 0.842, 0.858)	(0.654, 0.761, 0.863)	(0.269, 0.430, 0.640)	(0.733, 0.778, 0.822)
Cm_5	(0.814, 0.884, 0.953)	(0.936, 0.968, 1.000)	(0.748, 0.787, 0.820)	(0.456, 0.557, 0.642)	(0.956, 0.978, 1.000)
Cm_6	(0.605, 0.651, 0.698)	(0.776, 0.794, 0.812)	(0.671, 0.708, 0.739)	(0.272, 0.324, 0.381)	(0.711, 0.756, 0.800)
Cm_7	(0.860, 0.930, 1.000)	(0.819, 0.840, 0.860)	(0.904, 0.953,1.000)	(0.703, 0.845, 1.000)	(0.911, 0.933, 0.956)
Cm_8	(0.767, 0.884, 1.000)	(0.856, 0.886, 0.915)	(0.772, 0.857, 0.940)	(0.457, 0.651, 0.884)	(0.778, 0.800, 0.822)
AI	(0.860, 0.930, 1.000)	(0.936, 0.968, 1.000)	(0.904, 0.953, 1.000)	(0.703, 0.845, 1.000)	(0.956, 0.978, 1.000)

Alternative	C_6	C_7	C_8	C_9
AAI	(0.287, 0.324, 0.372)	(0.467, 0.500, 0.538)	(0.508, 0.526, 0.545)	(0.462, 0.490, 0.520)
Cm_1	(0.287, 0.324, 0.372)	(0.750, 0.808, 0.875)	(0.882, 0.938, 1.000)	(0.462, 0.490, 0.520)
Cm_2	(0.894, 0.944, 1.000)	(0.618, 0.677, 0.750)	(0.682,0.698, 0.714)	(0.597, 0.627, 0.660)
Cm_3	(0.361, 0.404, 0.459)	(0.583, 0.656, 0.750)	(0.517, 0.545, 0.577)	(0.856, 0.921, 0.998)
Cm_4	(0.459, 0.514, 0.583)	(0.512, 0.538, 0.568)	(0.508, 0.526, 0.545)	(0.718, 0.775, 0.843)
Cm_5	(0.361, 0.404, 0.459)	(0.778, 0.875, 1.000)	(0.600, 0.612, 0.625)	(0.869, 0.923, 0.984)
Cm_6	(0.287, 0.324, 0.372)	(0.656, 0.778, 0.955)	(0.833, 0.909, 1.000)	(0.644, 0.682, 0.725)
Cm_7	(0.361, 0.404, 0.459)	(0.467, 0.500, 0.538)	(0.600, 0.625, 0.652)	(0.887, 0.940, 1.000)
Cm_8	(0.361, 0.404, 0.459)	(0.618, 0.677, 0.750)	(0.652, 0.698, 0.750)	(0.729, 0.772, 0.821)
AI	(0.894, 0.944, 1.000)	(0.778, 0.875, 1.000)	(0.882, 0.938, 1.000)	(0.887, 0.940, 1.000)

Supplementary Table S3 Weighted normalized fuzzy decision matrix

Alternative	C_1	C_2	C_3	C_4	C_5
AAI	(0.100, 0.118, 0.136)	(0.110, 0.114, 0.118)	(0.033, 0.038, 0.042)	(0.005, 0.007, 0.010)	(0.098, 0.101, 0.105)
Cm_1	(0.106, 0.124, 0.142)	(0.125, 0.129, 0.132)	(0.033, 0.038, 0.042)	(0.005, 0.007, 0.010)	(0.105, 0.108, 0.111)
Cm_2	(0.100, 0.118, 0.136)	(0.110, 0.114, 0.118)	(0.036, 0.041, 0.045)	(0.005, 0.008, 0.011)	(0.098, 0.101, 0.105)
Cm_3	(0.183, 0.207, 0.230)	(0.121, 0.124 0.127)	(0.059, 0.065, 0.070)	(0.026, 0.036, 0.048)	(0.132, 0.135, 0.138)
Cm_4	(0.159, 0.189, 0.219)	(0.125, 0.127, 0.130)	(0.050, 0.058, 0.066)	(0.016, 0.026, 0.039)	(0.111, 0.118, 0.125)
Cm_5	(0.207, 0.224, 0.242)	(0.141, 0.146, 0.151)	(0.057, 0.060, 0.062)	(0.028, 0.034, 0.039)	(0.145, 0.149, 0.152)
Cm_6	(0.154, 0.165, 0.177)	(0.117, 0.120, 0.123)	(0.051, 0.054, 0.056)	(0.017, 0.020, 0.023)	(0.108, 0.115, 0.122)
Cm_7	(0.219, 0.236,0.254)	(0.124, 0.127, 0.130)	(0.069, 0.072, 0.076)	(0.043, 0.052, 0.061)	(0.138, 0.142, 0.145)
Cm_8	(0.195, 0.224, 0.254)	(0.129, 0.134, 0.138)	(0.059, 0.065, 0.071)	(0.028, 0.040, 0.054)	(0.118, 0.122, 0.125)
AI	(0.219, 0.236, 0.254)	(0.141, 0.146, 0.151)	(0.069, 0.072, 0.076)	(0.043, 0.052, 0.061)	(0.145, 0.149, 0.152)

Alternative	C_6	C_7	C_8	C_9
AAI	(0.008, 0.009, 0.010)	(0.047, 0.050, 0.054)	(0.039, 0.040, 0.041)	(0.048, 0.050, 0.054)
Cm_1	(0.008, 0.009, 0.010)	(0.075, 0.081, 0.088)	(0.067, 0.071, 0.076)	(0.048, 0.050,0.054)
Cm_2	(0.024, 0.025, 0.027)	(0.062, 0.068, 0.075)	(0.052, 0.053, 0.054)	(0.061, 0.065, 0.068)
Cm_3	(0.010, 0.011, 0.012)	(0.058, 0.066, 0.075)	(0.039, 0.041, 0.044)	(0.088, 0.095, 0.103)
Cm_4	(0.012, 0.014, 0.016)	(0.051, 0.054, 0.057)	(0.039, 0.040, 0.041)	(0.074, 0.080, 0.087)
Cm_5	(0.010, 0.011, 0.012)	(0.078, 0.088, 0.100)	(0.046, 0.047, 0.048)	(0.090, 0.095, 0.101)
Cm_6	(0.008, 0.009, 0.010)	(0.066, 0.078, 0.095)	(0.063, 0.069, 0.076)	(0.066, 0.070, 0.075)
Cm_7	(0.010, 0.011, 0.012)	(0.047, 0.050, 0.054)	(0.046, 0.048, 0.050)	(0.091, 0.097, 0.103)
Cm_8	(0.010, 0.011, 0.012)	(0.062, 0.068, 0.075)	(0.050, 0.053, 0.057)	(0.075, 0.080, 0.085)
AI	(0.024, 0.025, 0.027)	(0.078, 0.088, 0.100)	(0.067, 0.071, 0.076)	(0.091, 0.097, 0.103)
